# Supplementary material for: A learned oral signal drives long-lasting thirst satiation
Source: bioRxiv. 2026 Oct 1:2026.09.25.754368. Preprint. [Version 1] doi: 10.64898/2026.09.25.754368 (PMC13648541; doi:10.64898/2026.09.25.754368)
Supplement: Supplementary file 1 [file NIHPP2026.09.25.754368v1-supplement-2.pdf]

bioRxiv preprint doi: https://doi.org/10.64898/2026.09.25.754368; this version posted October 1, 2026. The copyright holder for this preprint (which was not certified by peer review) is the author/funder, who has granted bioRxiv a license to display the preprint in perpetuity. It is made available under a CC-BY 4.0 International license.

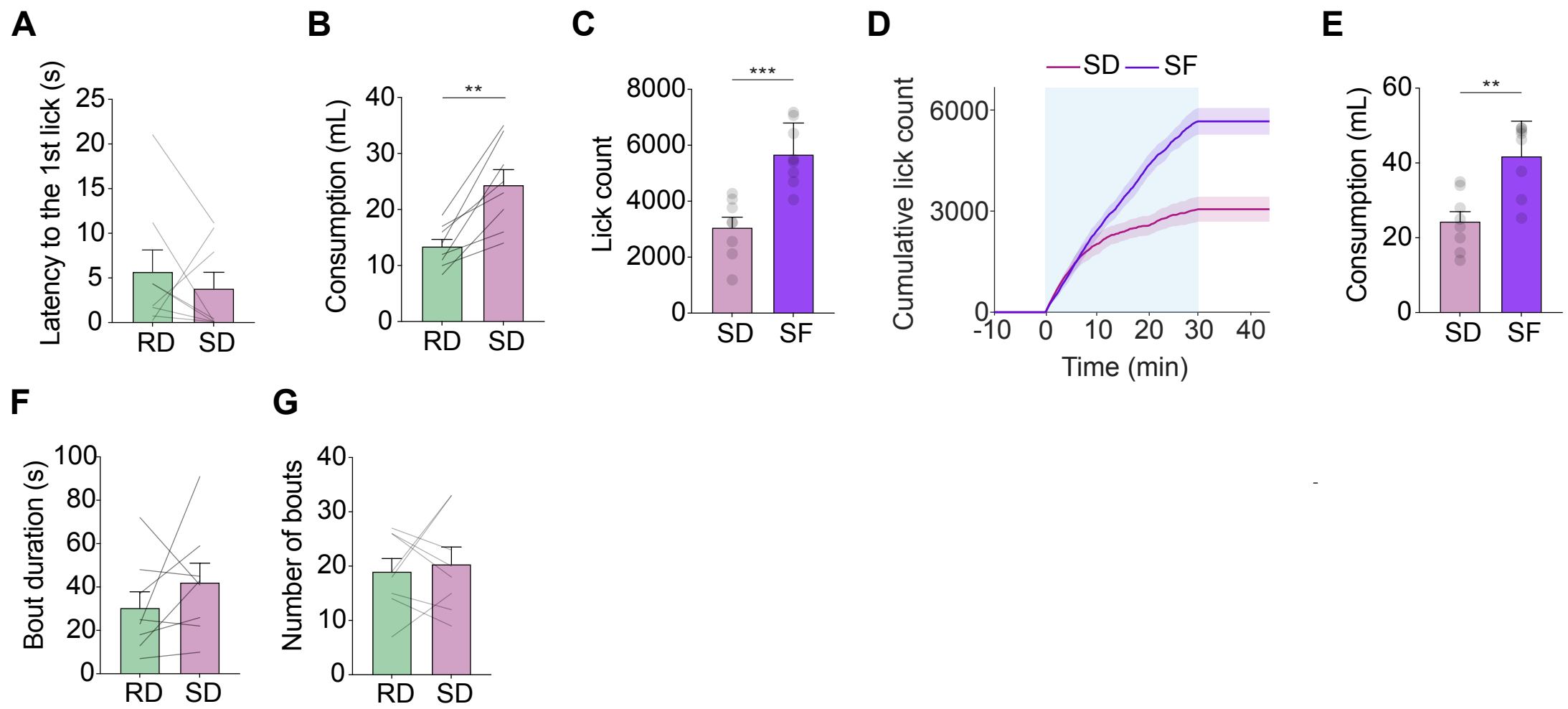

Figure S1 (related to Fig. 1) | Comparisons between consumption during real drinking, sham drinking, and sham feeding of sucrose

A, Latency from water access onset to the first lick during real drinking (RD) versus sham drinking (SD). **B,** Water consumption during RD versus SD. **C,** Total lick count during SD versus sham feeding (SF) of 0.8 M sucrose. **D,** Cumulative lick count during SD and SF. **E,** Fluid consumption during SD and SF. **F,** Average bout duration during RD and SD. **G,** Number of bouts during RD and SD. Sample sizes: n = 8 rats. $**p < 0.01$, $***p < 0.001$. All values are reported as mean ± SEM. More detailed statistical information is in **Table S1.**

bioRxiv preprint doi: https://doi.org/10.64898/2026.09.25.754368; this version posted October 1, 2026. The copyright holder for this preprint (which was not certified by peer review) is the author/funder, who has granted bioRxiv a license to display the preprint in perpetuity. It is made available under a CC-BY 4.0 International license.

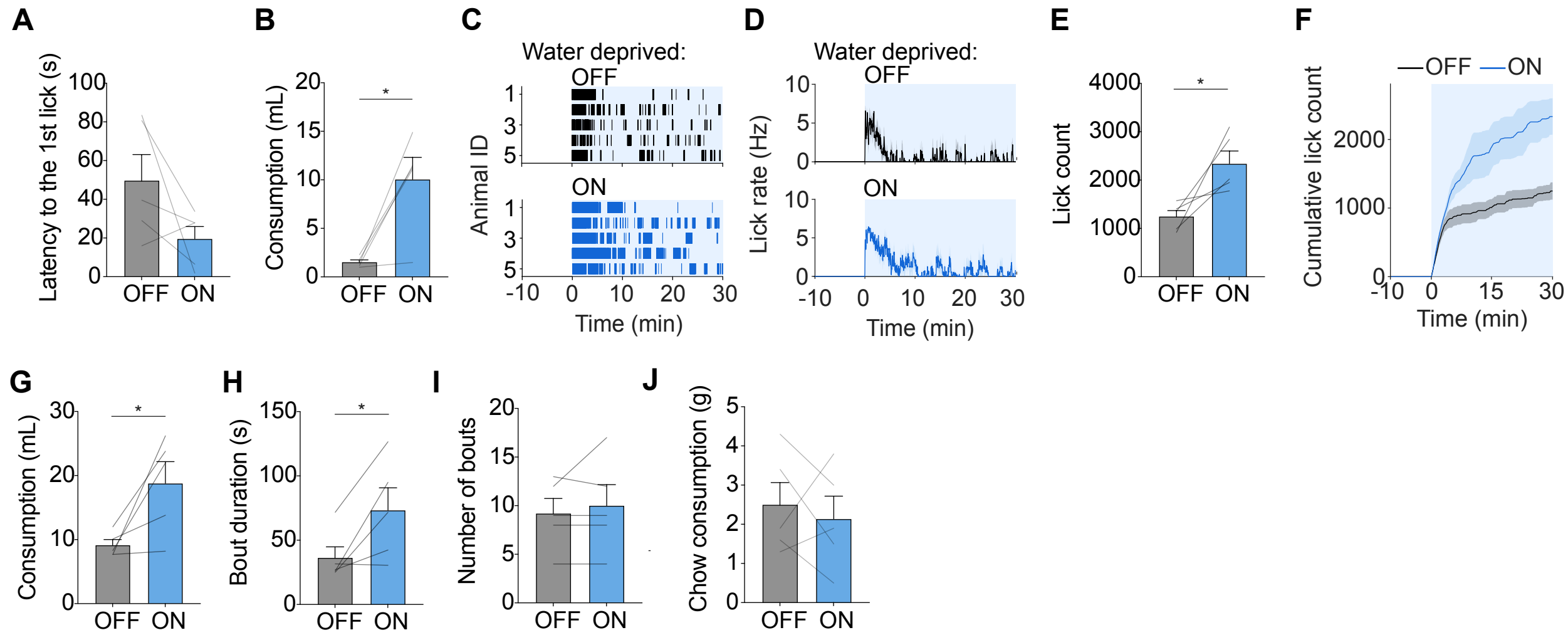

Figure S2 (related to Fig. 2) | Additional optogenetic functional validation experiments
A, Latency from water access onset to the first lick during laser-OFF versus laser-ON sessions. **B,** Water consumption by sated rats during laser-OFF versus laser-ON sessions. **C,** Lick rasters from water-deprived rats during 30-min water access in laser-OFF and laser-ON sessions. **D,** Lick rate during 30-min sessions in water-deprived rats during laser-OFF and open-loop laser-ON sessions. **E,** Total lick count in each session. **F,** Cumulative lick count in each session. **G,** Water consumption by water-deprived rats during laser-OFF versus laser-ON sessions. **H,** Average bout duration in each session. **I,** Number of bouts in each session. **J,** Chow consumption during 30-min sessions in hungry rats during laser-OFF and open-loop laser-ON sessions. Sample size: n = 5 rats. *$p < 0.05$. All values are reported as mean ± SEM. More detailed statistical information is in **Table S1.**

bioRxiv preprint doi: https://doi.org/10.64898/2026.09.25.754368; this version posted October 1, 2026. The copyright holder for this preprint (which was not certified by peer review) is the author/funder, who has granted bioRxiv a license to display the preprint in perpetuity. It is made available under a CC-BY 4.0 International license.

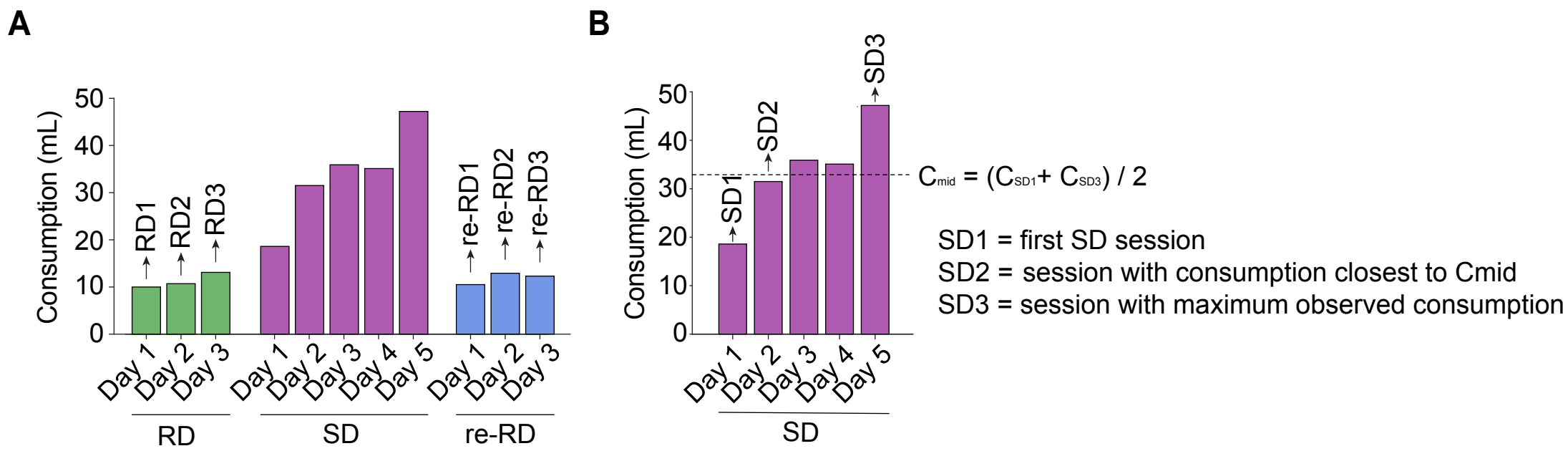

Figure S3 (related to Fig. 5) | Selection of three sessions for repeated RD, SD, and re-RD analyses in the water-drinking paradigm. **A,** Consumption across consecutive real drinking (RD), sham drinking (SD), and re-RD sessions from a representative animal. Three RD sessions were performed before SD, and three re-RD sessions were performed after SD; all RD and re-RD sessions were included in subsequent analyses. **B,** Behavioral alignment of SD sessions. Because the rate of behavioral adaptation during repeated SD varied across animals, three SD sessions were selected based on consumption to represent comparable early, intermediate, and late stages of adaptation, rather than using fixed chronological sessions. SD1 was defined as the first SD session and SD3 as the session with maximum observed consumption. The midpoint consumption was calculated as $C_{mid} = (C_{SD1} + C_{SD3}) / 2$, and SD2 was defined as the intervening session with consumption closest to Cmid. This procedure yielded three behaviorally aligned stages representing early, intermediate, and late SD adaptation for comparison with the three selected RD and re-RD sessions.

bioRxiv preprint doi: https://doi.org/10.64898/2026.09.25.754368; this version posted October 1, 2026. The copyright holder for this preprint (which was not certified by peer review) is the author/funder, who has granted bioRxiv a license to display the preprint in perpetuity. It is made available under a CC-BY 4.0 International license.

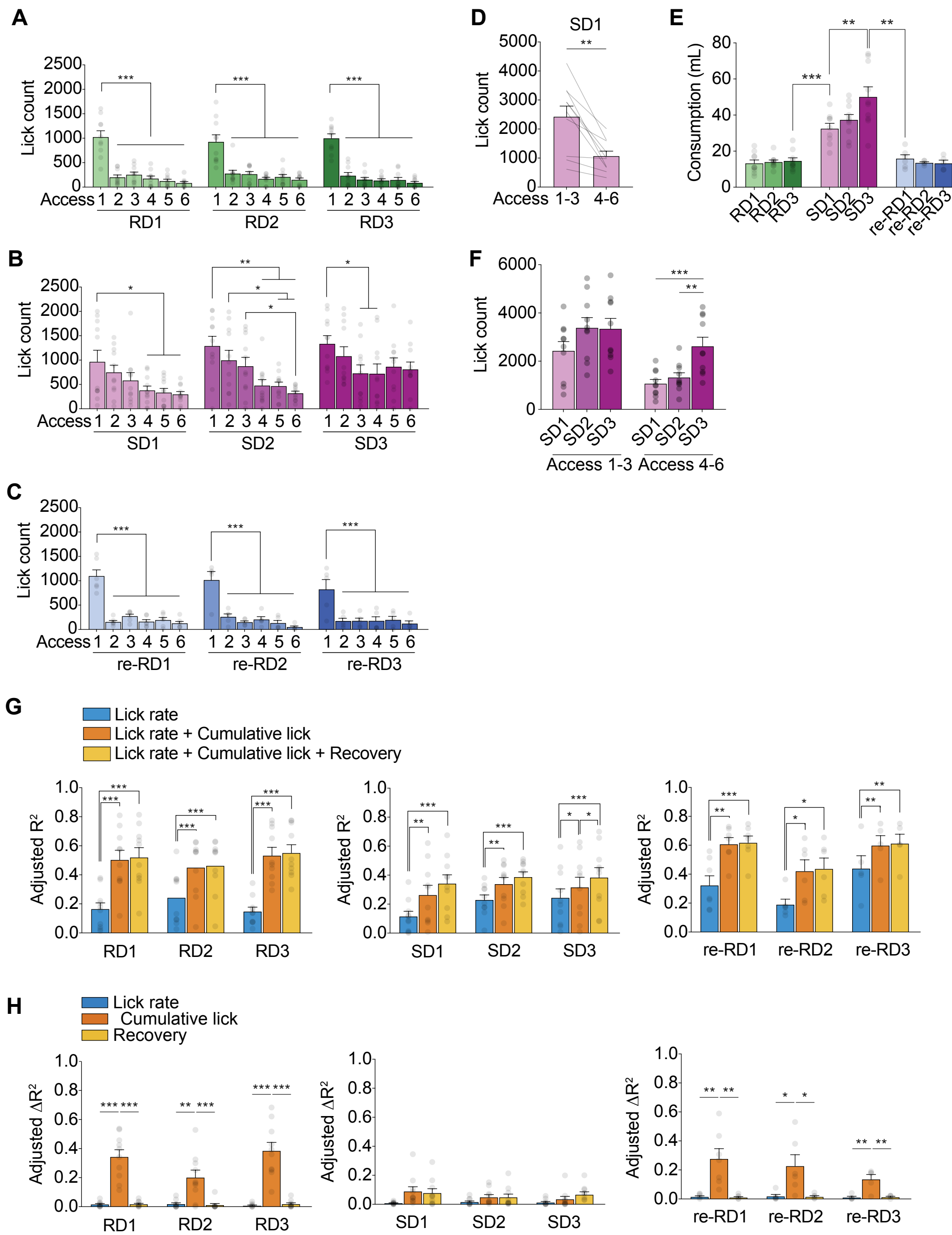

Figure S4 (related to Fig. 5) | Behavioral adaptation across repeated real and sham drinking sessions and GLM decomposition of MnPOAgtr1a activity

A-C, Lick counts during each of the six access periods across repeated real drinking (**A**; RD1-RD3), sham drinking (**B**; SD1-SD3), and return to real drinking (**C**; re-RD1-re-RD3) sessions. **D,** Lick counts during the first versus second half of the first sham-drinking session (SD1), quantified as the summed licks during Accesses 1-3 and Accesses 4-6, respectively. **E,** Total water consumption across repeated RD, SD, and re-RD sessions. **F,** Lick counts during the first three (Accesses 1-3) and last three (Accesses 4-6) access periods across repeated SD sessions. **G,** GLM performance for nested models incorporating lick rate alone, lick rate plus cumulative lick count, or lick rate plus cumulative lick count and post-access recovery, shown across repeated RD, SD, and re-RD sessions. Values indicate adjusted R^2 for each model. **H,** Unique contributions of the individual lick-rate, cumulative-lick, and post-access-recovery regressors to MnPO^{Agtr1a} activity across repeated RD, SD, and re-RD sessions, quantified as adjusted ΔR^2. Sample sizes: **A–B**, **D**, and **F**, n = 10 rats; **C**, re-RD1 n = 6 rats and re-RD2–re-RD3 n = 5 rats; **E**, RD1–RD3 n = 10 rats, SD1 n = 9 rats, SD2–SD3 n = 10 rats, re-RD1 n = 6 rats, and re-RD2–re-RD3 n = 5 rats; **G–H**, RD1–RD3 and SD1–SD3 n = 10 rats, re-RD1 n = 6 rats, and re-RD2–re-RD3 n = 5 rats. **p* < 0.05, ***p* < 0.01, ****p* < 0.001. All values are reported as mean ± SEM. More detailed statistical information is in **Table S1.**

bioRxiv preprint doi: https://doi.org/10.64898/2026.09.25.754368; this version posted October 1, 2026. The copyright holder for this preprint (which was not certified by peer review) is the author/funder, who has granted bioRxiv a license to display the preprint in perpetuity. It is made available under a CC-BY 4.0 International license.

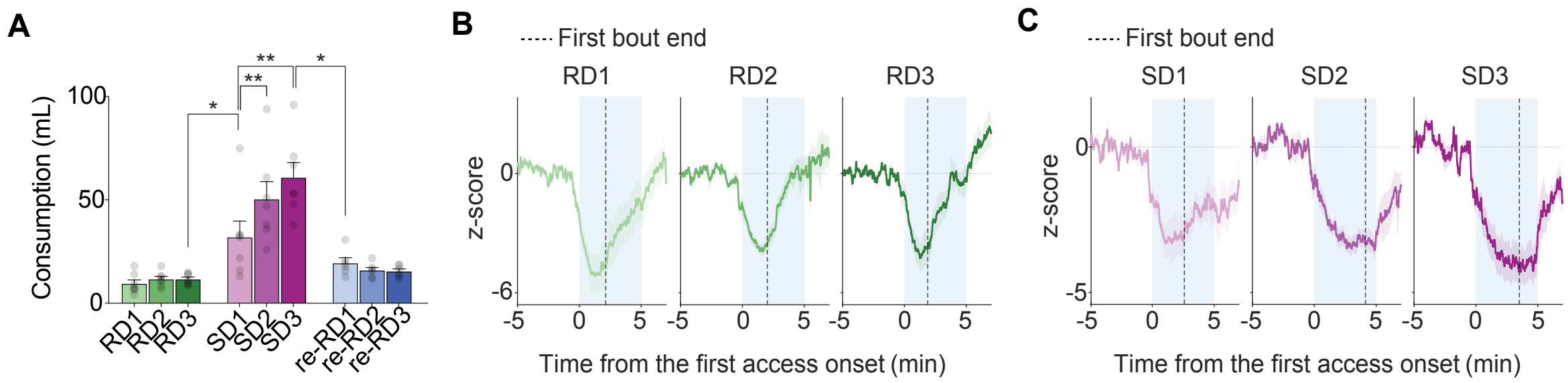

Figure S5 (related to Fig. 7) | Experience-dependent changes in consumption of hypertonic saline (0.3 M NaCl) and MnPO^{Agtr1a} recovery during hypertonic saline drinking

A, Consumption of hypertonic saline (0.3 M NaCl) across repeated real drinking (RD1-RD3), sham drinking (SD1-SD3), and return-to-real-drinking (re-RD1-re-RD3) sessions. **B,** Average MnPO^{Agtr1a} activity traces during repeated real drinking of hypertonic saline (RD1-RD3), aligned to the onset of the first access period. The vertical dashed line indicates the mean time of first lick-bout termination. **C,** Average MnPO^{Agtr1a} activity traces during the first access period across repeated sham-drinking sessions (SD1-SD3), aligned to the onset of the first access period. The vertical dashed line marks the mean time of first lick-bout termination. Sample sizes: **A**, RD1-RD3 and SD1-SD3 n = 8 rats and re-RD1-re-RD3 n = 7 rats; **B-C**, n = 8 rats. *$p < 0.05$, **$p < 0.01$. All values are reported as mean ± SEM. More detailed statistical information is in **Table S1.**
